# Evaluation of Polymeric Matrix Loaded with Melatonin for Wound Dressing

**DOI:** 10.3390/ijms22115658

**Published:** 2021-05-26

**Authors:** Beata Kaczmarek-Szczepańska, Justyna Ostrowska, Justyna Kozłowska, Zofia Szota, Anna A. Brożyna, Rita Dreier, Russel J. Reiter, Andrzej T. Slominski, Kerstin Steinbrink, Konrad Kleszczyński

**Affiliations:** 1 Department of Biomaterials and Cosmetics Chemistry, Faculty of Chemistry, Nicolaus Copernicus University, Gagarin 7, 87-100 Toruń, Poland; beata.kaczmarek@umk.pl (B.K.-S.); 285488@stud.umk.pl (J.O.); justynak@umk.pl (J.K.); 2 Department of Human Biology, Institute of Biology, Faculty of Biological and Veterinary Sciences, Nicolaus Copernicus University, Lwowska 1, 87-100 Toruń, Poland; zofia.szota@doktorant.umk.pl (Z.S.); anna.brozyna@umk.pl (A.A.B.); 3 Institute of Physiological Chemistry and Pathobiochemistry, Waldeyerstraße 15, 48149 Münster, Germany; dreierr@uni-muenster.de; 4 Department of Cellular and Structural Biology, UT Health Science Center, San Antonio, TX 78229, USA; reiter@uthscsa.edu; 5 Comprehensive Cancer Center, Department of Dermatology, University of Alabama at Birmingham, Birmingham, AL 35294, USA; aslominski@uabmc.edu; 6 Pathology and Laboratory Medicine Service, VA Medical Center, Birmingham, AL 35294, USA; 7 Department of Dermatology, University of Münster, Von-Esmarch-Str. 58, 48149 Münster, Germany; kerstin.steinbrink@ukmuenster.de

**Keywords:** melatonin, scaffolds, biopolymers, collagen, chitosan, cutaneous cells, wound healing

## Abstract

The development of scaffolds mimicking the extracellular matrix containing bioactive substances has great potential in tissue engineering and wound healing applications. This study investigates melatonin—a methoxyindole present in almost all biological systems. Melatonin is a bioregulator in terms of its potential clinical importance for future therapies of cutaneous diseases. Mammalian skin is not only a prominent melatonin target, but also produces and rapidly metabolizes the multifunctional methoxyindole to biologically active metabolites. In our methodology, chitosan/collagen (CTS/Coll)-contained biomaterials are blended with melatonin at different doses to fabricate biomimetic hybrid scaffolds. We use rat tail tendon- and *Salmo salar* fish skin-derived collagens to assess biophysical and cellular properties by (*i*) Fourier transform infrared spectroscopy—attenuated total reflectance (FTIR–ATR), (*ii*) thermogravimetric analysis (TG), (*iii*) scanning electron microscope (SEM), and (*iv*) proliferation ratio of cutaneous cells in vitro. Our results indicate that melatonin itself does not negatively affect biophysical properties of melatonin-immobilized hybrid scaffolds, but it induces a pronounced elevation of cell viability within human epidermal keratinocytes (NHEK), dermal fibroblasts (NHDF), and reference melanoma cells. These results demonstrate that this indoleamine accelerates re-epithelialization. This delivery is a promising technique for additional explorations in future dermatotherapy and protective skin medicine.

## 1. Introduction

The skin is a barrier organ that separates the body from the environment, protecting against microbial, physical, and chemical insults. In addition, primary skin disease and systemic disorders with associated cutaneous symptoms can result in pathological skin lesions, such as erosions, ulcers, and chronic wounds. Although its treatment includes the removal of mechanical influences and bandages with appropriate anti-inflammatory and antibacterial agents, amputation of the limb is still very often inevitable. Melatonin (*N*-acetyl-5-methoxytryptamine) is a ubiquitous physiological mediator that exists throughout the evolutionary scale of animals, plants, and unicellular organisms [1,2,3,4,5]. In mammals, it is most often characterized as a natural neurohormone synthesized in the pineal gland, from which it is released to modulate circadian rhythms [4]. In addition, many other tissues, and cells, including bone marrow [6], lymphocytes [7], retina [8], astrocytes [9], thymus [10], skin [11,12,13], and female reproductive organs (granulosa cells, cumulus cells, and oocytes) [14], synthesize melatonin. There is also evidence that melatonin is present in follicular fluid [15], and it is synthesized by oocytes [16,17]. Physiologically, melatonin functions as a multifaceted endogenous free radical scavenger and as a broad-spectrum antioxidant, and due to its amphiphilic nature, it can easily reach all cellular compartments [18,19,20]. Indeed, melatonin is ubiquitously localized in the cytosolic, membranous, mitochondrial, and nuclear compartments of the cell [21,22]. The highest melatonin concentrations are found in mitochondria [23,24], raising the possibility of functional significance for the involvement in mitochondrial activities [25,26]. For instance, most apoptotic signals originate in the mitochondria, and melatonin has well-known anti-apoptotic [27,28,29], anti-inflammatory [30], pro-differentiation [31,32,33], and oncostatic effects [21,22,23,24,25,26,27,28,29,30,31,32,33,34,35,36]. Melatonin acts through two major pathways: A receptor-mediated pathway (membrane, cytosolic, and nuclear receptors) and a receptor-independent pathway [37,38,39]. The receptor-mediated pathway is characterized by activating two types of membrane-specific receptors: the ML1 receptors, including MT1 (or Mel1a) and MT2 (or Mel1b) receptors, and the ML2 receptors, also called MT3 receptors. MT1 and MT2 are high-affinity receptors for melatonin with 60% homology, and their activation leads to an inhibition of the adenylate cyclase in target cells [37]. These G-protein-coupled receptors have mainly a role in the regulation of vigilance states, sleep/wake rhythms, and bone mass regulation [40,41]. Besides, acting through its receptors MT1 and MT2, melatonin activates multiple signaling pathways to modulate the activities and levels of several pivotal proteins in terms of scavenging free radicals. Melatonin, because of its electron transferability, is also engaged in complex repairing systems of damaged biomolecules. Namely, it effectively protects neurons and glial cells from Aβ-induced neurotoxicity and oxidative stress [38,42,43]. Thus, melatonin administration could reduce Aβ accumulation and enhance cognitive function against neurodegenerative progression. In other words, emerging findings are revealing that the decreased melatonin production in aged persons is considered an important factor for developing Alzheimer’s disease (AD) [38,44,45]. The above features prompted us to examine the application of melatonin administered using chitosan/collagen scaffolds to discover its “undiscovered” potential with regard to wound healing.

Skin wounds are induced by thermal or electrical factors, but also by chemicals and UV radiation. The most common burns are those triggered by thermal pulses, which result in tissue damage and inflammation at the wound site. Further consequences of burns occur at the cellular stage, where reactive oxygen species (ROS) and reactive nitrogen species (RNS) are massively formed. Classic wound treatment methods based on 1% silver sulfadiazine are not sufficient because they exhibit cytotoxic activity and delay the wound healing process. The use of nanogel dressings with high water content, in combination with bioactive melatonin molecules, accelerates the healing and regeneration process of the skin. Enrichment of the composite with natural polysaccharides, such as chitosan and hyaluronic acid, guarantees the ability to absorb liquids, maintains a moist environment, and enables gas exchange. Nevertheless, the biomedical properties that favor biocompatibility and biodegradability of the described composites are also beneficial.

Hydrogels based on hyaluronic acid, chitosan, and melatonin have been in part characterized, and they have excellent physicochemical and antimicrobial properties, which support the wound healing processes and ensure biocompatibility with the skin [46]. For example, Chen et al. [47] tested the injectable melatonin-loaded carboxymethyl chitosan (CMCS)-based hydrogel, and they assumed that it induces granulation tissue formation and accelerates wound healing. Qian et al. [48] fabricated a 3D melatonin/polycaprolactone nerve guide conduit. These materials freely exchange nutrients and support long-term structural stability. Thereby, melatonin/polycaprolactone materials may find application in nerve tissue engineering. Xu et al. [49] reported that melatonin is a bioactive substance that can effectively promote muscle recovery by inhibiting oxidative stress and inflammation. Also, 3D-printed *β*-tricalcium phosphate (*β*-TCP) scaffolds blended with melatonin were studied for bone regeneration [50]. Bone mesenchymal stem cells have shown great viability and proliferation in this type of scaffold. Manjunath et al. [51] synthesized melatonin-loaded albumin nanoparticles and entrapped them into a polycaprolactone scaffold. Such modification increased the therapeutic potential of the scaffolds for cartilage regeneration. Herein, our studies characterized scaffolds based on natural polymers loaded with melatonin as an active substance for tissue regeneration processes.

## 2. Results

### 2.1. Fourier Transform Infrared Spectroscopy—Attenuated Total Reflectance (FTIR–ATR)

**Figure 1:**
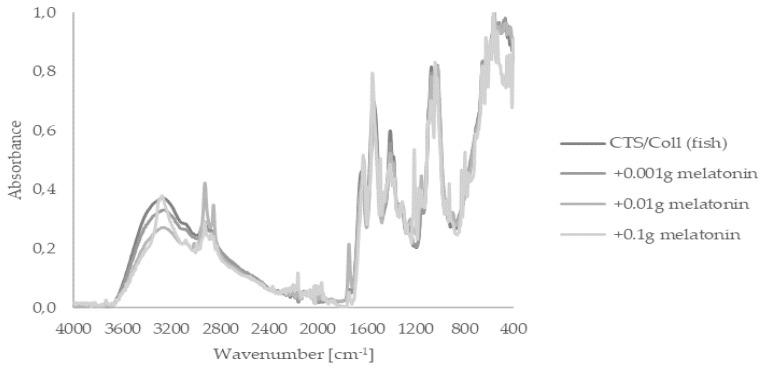
The FTIR-ATR spectra of scaffolds based on chitosan (CTS)/collagen (Coll) (derived from Salmo salar fish skin) mixture with and without melatonin in a dose-dependent manner.

**Figure 2:**
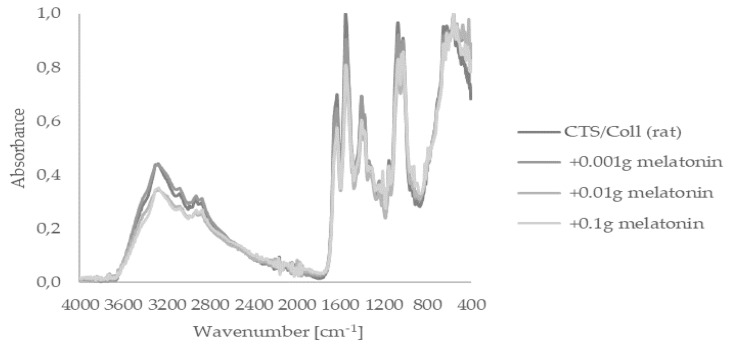
The FTIR-ATR spectra of scaffolds based on chitosan (CTS)/collagen (Coll) (derived from rat tail tendon) mixture with and without melatonin in a dose-dependent manner.

FTIR-ATR spectroscopy is a fast, nondestructive, noninvasive, label-, and reagent-free, inexpensive, sensitive, and highly reproducible physicochemical tool for the characterization of polymers. Firstly, we focused on the assessment of the presence of functional groups within melatonin-enriched chitosan (CTS)/collagen (Coll). Thus, applied FTIR-ATR analysis of subjected materials sourced from fish or rats showed spectra peaks indicating their characteristic bands, i.e., I, II, and III (Figure 1 and Figure 2). Fish-derived collagen revealed peaks at 1645 (Band I), 1554 (Band II), and 1263 cm^−1^ (Band III). In agreement with a previous study [52], strong peaks within the range of 3665–2328 cm^−1^ referred to –NH, –OH, or Amide A from collagen, were observed. A similar pattern was noticed within rat-derived collagen presented in Figure 2. It should still be stated that Amide bands themselves are sensitive to the secondary structure of the protein; however, the Amide I-III region is similar for both types of collagen. Thereby, we assume that changes in Amide A shape are triggered by the preparation method instead of changes in collagen secondary structure. Both types of collagen, either fish or rat, did not present any differences in FTIR-ATR spectra for materials containing melatonin or without this indoleamine.

### 2.2. Scanning Electron Microscope (SEM)

**Figure 3:**
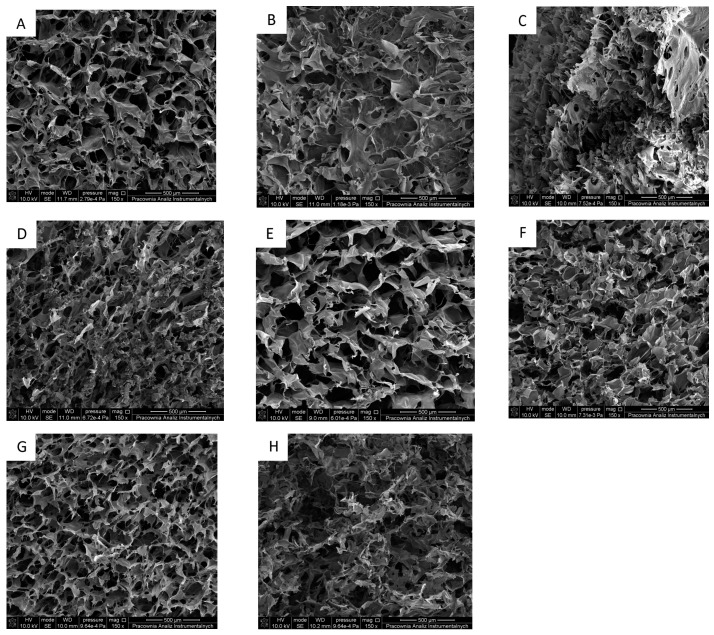
Scanning electron microscopy (SEM) images of CTS/Coll (fish) (**A**–**D**): (**A**) Without melatonin, (**B**) +0.001 g melatonin, (**C**) +0.01 g melatonin, (**D**) +0.1 g melatonin; and CTS/Coll (rat) (**E**–**H**): (**E**) Without melatonin, (**F**) +0.001 g melatonin, (**G**) +0.01 g melatonin, (**H**) +0.1 g melatonin.

SEM enables a clear observation of structures. For scaffolds, the shape and connectivity of pores may be detected. Collected results are in line with scanning electron microscopy. In these analyses, it was visible that the liophilization process allowed to obtain 3D porous structures with open interconnected pores (Figure 3). Thus, this phenomenon is crucial for designed materials as it allows for gas exchange via the dressing material, increasing the effectiveness of wound healing [53].

### 2.3. Thermogravimetric Analysis (TG)

Thermogravimetric analysis (TGA) is an analytical technique used to determine a material’s thermal stability and its fraction of volatile components by monitoring the weight change that occurs as a sample is heated at a constant rate. The next step was the assessment of water loss by resultant materials (Table 1) using gradual thermogravimetric assay where the first (T_1_) and the second (T_2_) stage indicate the loss of structural bound water, while the third one (T_3_) refers to the degradation of the polymeric chain [54].

Obtained results revealed that temperature within T_1_ and T_2_ for CTS/Coll (fish) are higher than blends composed from CTS/Coll (rat). Nevertheless, T_3_ inverted this trend towards rat-derived collagen, reaching 292.28 °C compared to CTS/Coll (fish) with 287.51 °C indicating different denaturation temperatures of collagens from different sources is in line with a previous report by Prus and Kozłowska [55]. Importantly, the addition of melatonin itself to the subjected scaffolds containing either fish- or rat-derived collagen did not affect their biophysical properties.

### 2.4. Cellular Assessments Using Cutaneous Models

**Figure 4:**
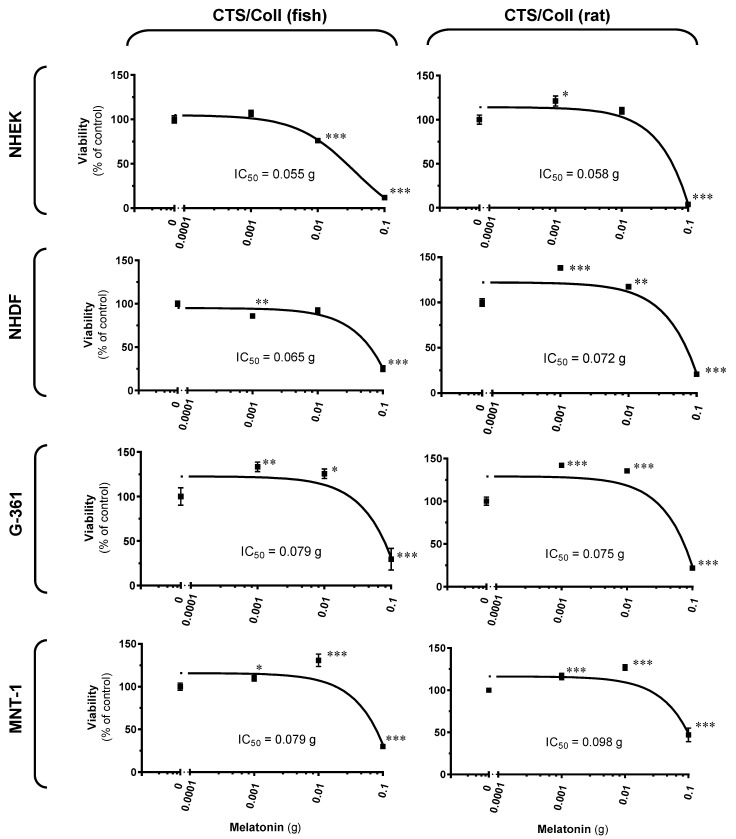
Human epidermal keratinocytes (NHEK), dermal fibroblasts (NHDF), as well as human amelanotic (G-361) and melanotic (MNT-1) melanoma cells, were seeded on CTS/Coll fish- or rat-derived scaffolds containing melatonin a in dose-dependent manner, cultured for 96 h, and viability was assessed using the MTT viability assay as described in Materials and Methods. Data are presented as mean +S.E.M. (*n* = 6), expressed as a percentage of the control cells (scaffold without melatonin), and IC_50_ values were determined accordingly. Statistically significant differences versus the control were indicated as * *p* < 0.05, ** *p* < 0.01, *** *p* < 0.001.

Herein, we wanted to assess the difference in cell growth on subjected melatonin-enriched scaffolds, which could mimic re-epitalization. Thus, evaluated viability of cutaneous cells with resultant IC_50_ values are presented in Figure 4, where human epidermal keratinocytes or dermal fibroblasts were tested in comparison to two human melanoma models, i.e., amelanotic (G-361) and melanotic (MNT-1) melanoma cell lines. Statistically significant enhancement was observed in cell proliferation, both, using fish or rat collagen. Namely, CTS/Coll (fish) containing melatonin triggered cell viability by 33% (0.001 g melatonin) or 26% (0.01 g melatonin) for G-361 cells, and a similar pattern of regulation was noticed for MNT-1 melanoma by 10% or 31%, respectively. Comparatively, CTS/Coll (rat) enriched by subjected melatonin enhanced cell proliferation ranging from 16% to 42% for melanoma cells and from 21% to 38% for keratinocytes/fibroblasts for scaffolds with 0.001 g melatonin. The addition of 0.01 g melatonin enhanced up to 35% and 17% for melanoma cells and human keratinocytes or fibroblasts, respectively. Interestingly, the highest dose of melatonin, i.e., 0.1 g led to a significant decrease of cell proliferation reaching its level versus the control sample ranging from 12% to 30% and from 4% to 47% for CTS/Coll (fish) and CTS/Coll (rat), respectively.

## 3. Discussion

Collagen a protein that has found wide-spread applications in medicine, due to its biocompatible and safety. It is obtained mainly from fungi, fish skin, scales, but also from the rat tail, pig or beef tissues, sea sponges, jellyfish, and egg capsules of the dogfish. Collagen isolated from different sources differs in terms of denaturation temperatures, e.g., approximately 33 °C and 39 °C for fish and rat collagen, respectively [56].

To date, the main disadvantage of collagen-based materials is their low stability in aqueous conditions. Thus, it dissolves quickly to limit its application in tissue regeneration purposes. Furthermore, it is necessary to mix collagen with other polymers and/or use so-called cross-linkers that react with collagen functional groups and improve collagen stability per se.

One of the methods to improve its biophysical properties is preparing collagen-based materials by mixing them with other macromolecules. Namely, collagen can be blended with other proteins, such as silk fibroin or elastin [57,58], but also with polysaccharides, including hyaluronic acid [59], chitosan [60], or sodium alginate [61]. Following an earlier study of Kaczmarek et al. [62], herein, we investigated different scaffolds based on collagen derived from rat tail tendon or from *Salmo salar* fish skin mixed with chitosan. Resultant matrices were assessed as carriers for melatonin. FTIR analysis was carried out to determine changes in the polymeric structure of collagen from those two different sources, as well as the impact of melatonin itself on its characteristics. The obtained FTIR spectra did not show any significant changes in the peaks’ profiles. Furthermore, characteristic peaks for collagen (Amide I, II, and III), as well as a typical peak corresponding to Amide A, hydroxyl, and amine groups, were observed. This is in line with our previous study, where we confirmed that the interaction between collagen and chitosan hydrogen occurs without new covalent formation [62]; the addition of melatonin did not influence its polymeric structure. It should, however, be noted that an increased number of hydroxyl groups in the resulting biomaterials determines elevated water binding. It is highly desirable that the moisture environment is constantly maintained, avoiding skin dehydration and scar formation during wound healing, as previously shown [63,64].

3D materials with highly porous structures are desired candidates for tissue regeneration where significant enhancement of the nutrient maintenance for targeted cutaneous cells is required. We also noticed that the resultant materials kept their shapes and homogeneity despite the addition of melatonin. Finally, the presence of indoleamine did not affect the thermal stability of the resultant scaffolds what is consistent with Andonegi et al. [65], where three decomposition stages of collagen/chitosan materials were assessed. Also, our observations are in line with the previous report of Correa et al. [66], in which melatonin exerted a positive impact on wound dressing by increased water entrapment, thereby substantially improving wound healing. The authors observed that the three-layered nanofiber wound dressing containing melatonin shows remarkable wound repair capacities by reducing the wound healing duration. Moreover, the authors carried out a histopathological evaluation in which showed the complete regeneration of the epithelial layer, remodeling of wounds, collagen synthesis, and reduction in inflammatory cells.

Comparative assessment using skin cells revealed that melatonin induced prominent differences in cell viability in a dose-dependent manner, with its highest concentration negatively affecting cell proliferation. It should be here added that our previous reports revealed that melatonin as well its metabolites are present within cutaneous cells, human epidermis, but also in human melanoma cell lines [12,31,36,67,68]. Nevertheless, lower doses of this indoleamine significantly enhanced this parameter, both, for human epidermal keratinocytes and dermal fibroblasts, but also for human melanoma cells used as reference cellular models. Elevated biophysical capacities of resultant scaffolds, as well as increased numbers of proliferating cells are in line with previous reports [69,70], in which melatonin did not impact cell viability, showed good stability characteristics, and could be safely applied, thereby improving wound healing potential.

## 4. Materials and Methods

### 4.1. Reagents

Chitosan (CTS; DD = 77%, M*v* = 5.4 × 10^5^ g/mol), Minimum Essential Medium Eagle (MEM) (1000 mg/L), 1% penicillin-streptomycin solution, 3-(4,5-dimethylthiazol-2-yl)-2,5-diphenyltetrazolium bromide (MTT), acetic acid, ethanol (EtOH), HEPES (1 M), HCl, isopropanol, melatonin, and non-essential amino acids (NEAA) (100×) were purchased from Sigma (St. Louis, MO, USA). Fetal bovine serum, 0.05% trypsin/0.53 mM EDTA solution, 1 × PBS (pH 7.4), *L*-glutamine (200 mM), AIM-V™ medium were purchased in Thermo Fisher Scientific (Waltham, MA, USA). Collagen (Coll) used in this study was sourced from *Salmon salar* fish skin [71], and isolated rat tail tendon along the procedures described previously [72,73].

### 4.2. Sample Preparation

Chitosan (CTS) and respective types of collagen (Coll) were dissolved in 0.1 M acetic acid, reaching the final solution of 1%. CTS/Coll was mixed in a 50/50 (*w/w*) ratio on the magnetic stirrer. Melatonin was dissolved in 3 drops of EtOH, filled by 0.1 M acetic acid to the final volume of 1 mL, and mixed together with CTS/Coll mixture. After that, the content was placed into 24-wells plates, frozen and lyophilized (ALPHA 1–2 LDplus, CHRIST, −20 °C, 100 Pa, 48 h). Each scaffold (2.5 g of CTS/Coll mixture) contained melatonin in a dose-dependent manner, i.e., 0.001 g, 0.01 g, and 0.1 g. Scaffold without melatonin was used as the control sample.

### 4.3. Fourier Transform Infrared Spectroscopy—Attenuated Total Reflectance (FTIR–ATR)

FTIR-ATR spectra were made for each type of scaffold in the range 4000–400 cm^−1^ by the spectrometer (Nicolet iS110) equipped with a diamond crystal with a resolution 4 cm^−1^. Spectra were taken with 64 scans.

### 4.4. Scanning Electron Microscope (SEM)

The morphology of the obtained scaffolds was studied using Scanning Electron Microscope (SEM; LEO Electron Microscopy Ltd., Cambridge, UK). SEM images have a resolution of 200 µm. Samples were covered by gold to form the conductive surface for the electron beam interaction.

### 4.5. Thermogravimetric Analysis (TG)

Thermogravimetric analysis (TG) was performed on a TA Instruments SDT 2960 Simultaneous TGA–DTA in nitrogen and at a heating rate of 10 °C/min and the heating program of 25–600 °C. Spectra were analyzed with the use of the TA Universal Analysis program.

### 4.6. Cell Culture

Normal human epidermal keratinocytes (NHEK) and normal human dermal fibroblasts (NHDF) were supplied by PromoCell (Heidelberg, Germany) and American Type Culture Collection (ATCC) (Manassas, VA, USA), respectively. NHEK were grown in Keratinocyte Growth Medium 2 supplemented with 1% penicillin-streptomycin solution. In comparison, NHDF were maintained in MEM medium supplemented with 10% (*v/v*) heat-inactivated fetal bovine serum, 2 mM *L*-glutamine, and 1% (*v/v*) streptomycin-penicillin solution. Comparatively, a human melanoma cell model was used, such as melanotic MNT-1 cells acquired as a gift from Dr. Cédric Delevoye (Institute Curie, Paris, France) and amelanotic G-361 cell line supplied by ATCC (Manassas, VA, USA). MNT-1 cells were cultured along modified culture medium content [74], i.e., MEM medium supplemented with 20% (*v/v*) heat-inactivated fetal bovine serum, 10% (*v/v*) AIM-V™ medium, 2 mM *L*-glutamine, 10 mM HEPES, 1% (*v/v*) NEAA, and 1% (*v/v*) streptomycin-penicillin solution. G-361 cells were maintained in MEM medium supplemented with 10% (*v/v*) heat-inactivated fetal bovine serum, 2 mM *L*-glutamine, and 1% (*v/v*) streptomycin-penicillin solution. Cells were seeded on 24-well plates at the density of 0.5 × 10^5^ cells/well and allowed them to attach to the surface of the subjected scaffolds for 24 h. After that, cells were cultured in supplemented culture medium in a humidified atmosphere of 5% CO_2_ at 37 °C for 96 h, while the culture medium was exchanged every 48 h. Differences in cell viability were assessed using the MTT assay.

### 4.7. Cell Viability Assay

MTT assay was conducted along with the previously described procedure [75]. MTT (5 mg/mL in 1 × PBS) was prepared in respective culture medium (the final dilution, 1:10), 100 μL of assay reagent was added to each well, and cells were subsequently incubated for 3 h in a humidified atmosphere of 5% CO_2_ at 37 °C. The resultant formazan crystals were dissolved using 100 μL isopropanol/0.04 N HCl, absorbance was measured at *λ* = 595 nm using the BioTek ELx808™ microplate reader (BioTek Instruments, Inc., Winooski, VT, USA), results were normalized to the control cells, and IC_50_ values were subsequently determined.

### 4.8. Statistical Analysis

Data were expressed as pooled means + standard error of the mean (S.E.M.) of six independent experiments (*n* = 6). Statistically significant differences between results were determined by the univariate analysis of variance (ANOVA) or the Student’s *t*-Test and appropriate post-hoc analysis using GraphPad Prism 7.05 software (La Jolla, CA, USA).

All the analyses are presented as a percentage of the control sample, and a *p* < 0.05 was considered statistically significant.

## 5. Conclusions

**Figure 5:**
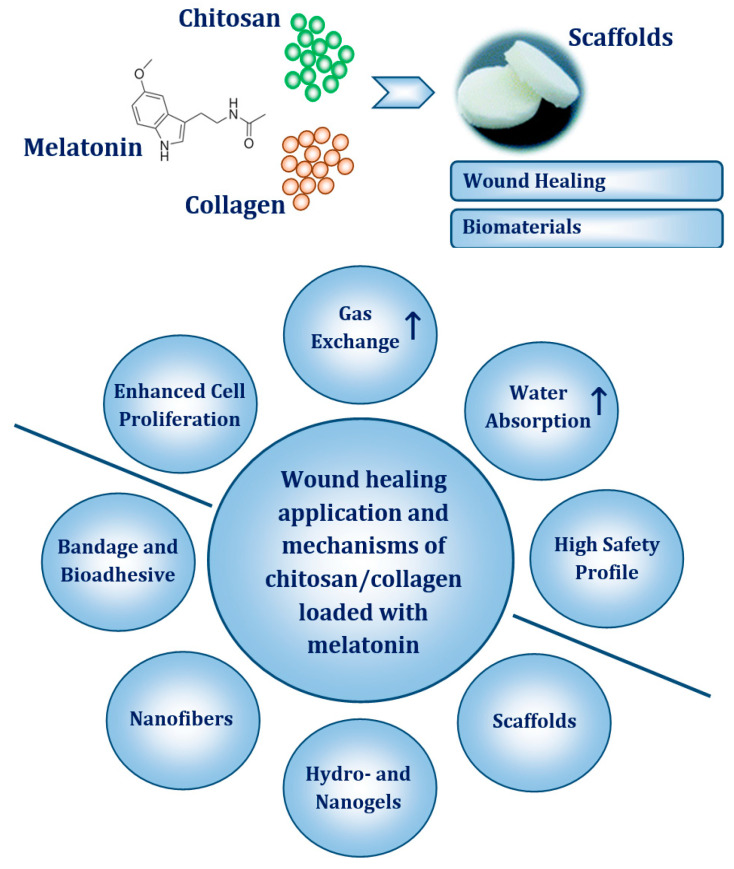
The most important mechanisms of chitosan/collagen matrices loaded with melatonin and its application in wound healing.

This study evaluated the comparatively biophysical properties of chitosan/collagen scaffolds containing melatonin as a potential additive in polymeric matrices. Our data provides new insight into a considerable improvement of wound dressing where subjected indoleamine accelerates wound healing potential considering its application and mechanisms, as presented in Figure 5. Given that melatonin is essentially nontoxic, readily available over the counter in different formulations, and that it meets the definition of a natural product, its topical and transepidermal delivery is a promising area for full exploration in future preventive and therapeutic approaches for skin tissue engineering and wound healing.

## Figures and Tables

**Table 1:** Parameters of thermal decomposition of scaffolds assessed along with the maximum temperature for obtained peaks based on TG-DTG spectra.

Specimen	T_1_ [°C]	T_2_ [°C]	T_3_ [°C]
*CTS/Coll (fish)*	61.23	190.43	287.51
+0.001 g melatonin	53.64	188.70	286.27
+0.01 g melatonin	58.42	175.31	289.51
+0.1 g melatonin	57.73	n.o.	289.57
*CTS/Coll (rat)*	60.19	175.54	292.28
+0.001 g melatonin	52.03	169.96	293.92
+0.01 g melatonin	58.08	171.76	291.45
+0.1 g melatonin	n.o.	n.o.	280.83

n.o.: peak not observed.
